# Analysis of Altitude Hypoxia Training and In-Flight Hypoxia Events among the Helicopter Aircrews

**DOI:** 10.3390/ijerph18168405

**Published:** 2021-08-09

**Authors:** Kwo-Tsao Chiang, Hsin Chu, Min-Yu Tu, You-Jin Lin, Sing-Hong Lin, Yu-Hsin Wen, Chung-Yu Lai

**Affiliations:** 1 Aviation Physiology Research Laboratory, Kaohsiung Armed Forces General Hospital Gangshan Branch, Kaohsiung City 820, Taiwan; charco66@gmail.com (K.-T.C.); du0807@yahoo.com.tw (M.-Y.T.); allenwen@msn.com (Y.-H.W.); 2 School of Public Health, National Defense Medical Center, Taipei City 114, Taiwan; 3 Medical Section, Civil Aviation Medical Center, Taipei City 105, Taiwan; hrchu@mail.ndmctsgh.edu.tw; 4 Graduate Institute of Aerospace and Undersea Medicine, National Defense Medical Center, Taipei City 114, Taiwan; youjinlin7@gmail.com; 5 Department of Health Business Administration, Meiho University, Pingtung County 912, Taiwan; 6 Department of Life Sciences and PhD Program in Translational Medicine, National Chung Hsing University, Taichung City 402, Taiwan; 7 Institute of Medical Science and Technology, National Sun Yat-sen University, Kaohsiung City 804, Taiwan; 8 Medical Affairs Section, Chief Inspector’s Office, Headquarters of Air Force, Taipei City 104, Taiwan; venox93@gmail.com

**Keywords:** helicopter aircrew, altitude hypoxia training, hypoxia, chamber flight, in-flight hypoxia

## Abstract

All aircrews are required to undertake the altitude hypoxia training and be familiarized with the hypobaric effect on their physiological regulation. Due to the characteristics of the helicopter aircrafts, few researches have reported in-flight hypoxia events among the helicopter aircrews. The main goal of this study was designed to compare the hypoxia symptoms of helicopter aircrews between the altitude hypoxia training and during flight. We developed a questionnaire to collect the details of chamber flights and in-flight hypoxia events in 2019. All data were managed by the SPSS software and two-tailed 0.05 alpha level was considered as a significant level. Of the 213 study participants, there were eight (3.8%) cases that experienced hypoxia symptoms during the flight. The top five symptoms that appeared both in the last and current altitude hypoxia trainings were visual impairment (20.7%), difficulty concentrating (12.7%), tiredness (12.2%), cognitive impairment (8.0%), and air hunger (5.2%). Meanwhile, the frequency of those symptoms above was not significantly different from the last or current training compared with those in-flight hypoxia events. The survey unveiled a series of consistency correlations of hypoxia symptoms between the chamber flights and in-flight environment for the helicopter aircrew group.

## 1. Introduction

The human body inhales the air into the lungs. The content of the atmosphere is essentially 21% oxygen which is a vital element for life support. Gas exchange happens in the alveoli based on the pressure gradient. However, partial pressure of oxygen decreases by the altitude ascent. If the diffusion rate of oxygen exchange is lower than the consumption rate of the human body due to the reduction of the pressure gradient, people will face the challenge of hypoxia problem when staying at a high-altitude environment [1].

As the military aircrafts have a higher performance ascent to high altitude, aircrews are bound to be threatened by the hypobaric hypoxia. It is generally accepted that the threshold in the aviation medicine is 3048 m (10,000 ft) (so-called physiological altitude) which flight performance is theoretically not impaired [1]. Even though the cabin pressurization and supplementary oxygen system has been already well-developed, in-flight hypoxia attacks that causally contributed to severe mishaps would sometimes be heard [2].

Some previous studies summarized that the main causes of in-flight hypoxia incidents were depressurized or failed pressurization, failure of the oxygen system, and the oxygen hose disconnected among the military fixed-wing aircraft aircrews [3,4]. After the onset of hypoxia, aircrews have a small period of time to perceive the ongoing event by personal hypoxia symptoms and take timely corrective steps before the incapacitation. Altitude hypoxia training by a hypobaric chamber or reduced oxygen breathing device (ROBD) have been conducted to strengthen the aircrews’ hypoxia awareness [5,6,7,8]. However, physiological responses and incidence of altitude illness by using a ROBD are different from the hypobaric chamber. ROBD was also constructed to improve the efficiency and safety of the hypoxia awareness training.

Altitude hypoxia training at the simulated altitude of 7620 m (25,000 ft) containing a regular refresher course is commonly conducted for military fixed-wing aircraft aircrews worldwide. After the training, aircrew members were less likely to lose their consciousness after another exposure of hypobaric environment [9]. Some reports noted that the main hypoxia symptoms including cognitive impairment, slowing response, visual changes, lightheadedness/dizziness, and hot flushes were not apparently different between the two training sessions based on the Environment Symptoms Questionnaire [10,11,12]. In addition, aircrews pointed out that in-flight hypoxia events that happened were quickly recognized in accordance with the unique personal symptoms and the sensation during altitude hypoxia training [3,5].

Compared with fixed-wing airframes, helicopter aircrafts are characterized by lack of cabin pressurization and usually without supplementary oxygen system. Although helicopter aircrews were directly exposed to the hypoxic environment during flight, they seldom flew over the physiological altitude [3048 m (10,000 ft)]. Anecdotally, some helicopter aircrews in Taiwan didn’t believe that in-flight hypoxia would happened to them. They, therefore, were reluctant about attending the 5486-m (18,000-ft) refresher altitude hypoxia training. However, in-flight hypoxia cases have been reported from the helicopter aircrews [13]. Until now, insufficient data were clarified by the relationship of hypoxia symptoms between the altitude hypoxia training and the in-flight hypoxia events. Rare studies have explored the consistency of hypoxia symptoms between the last and current trainings among the helicopter aircrews.

The purposes of this current survey are as follow; (1) to describe the main hypoxia symptoms during the altitude hypoxia training, (2) to calculate the incident rate of in-flight hypoxia events, and (3) to examine the consistency of hypoxia symptoms between the last and current altitude hypoxia trainings and in-flight hypoxia events among the helicopter aircrews. The findings could also be used to emphasize the value of regular altitude hypoxia training on the flight safety among the helicopter aircrews.

## 2. Materials and Methods

### 2.1. Subjects

Military helicopter aircrews are required to undertake 5486-m (18,000-ft) altitude hypoxia training to enhance their memory every four years based on Taiwan’s regulation. We recruited 213 study subjects from the military helicopter aircrews who received refresher training in Aviation Physiology Research Laboratory (APRL). Meanwhile, all of them have be qualified by annual health examination and obtained the medical clearance signed by a flight surgeon before the altitude hypoxia training. This protocol was approved by the Kaohsiung Armed Forces General Hospital Institutional Review Board in Kaohsiung City, Taiwan (No. KAFGH 107-017).

### 2.2. Equipment

Altitude hypoxia training was conducted in the APRL hypobaric chamber (Contract 540, Guardite Inc., Chicago, IL, USA). The training capacity of this hypobaric chamber is 16 trainees inside the main chamber by using a vacuum pump to replicate the low pressure environment at ground level. Military helicopter aircrews receive the 5486-m (18,000-ft) refresher altitude hypoxia training every four years. During the training, aircrews will (1) experience the mechanical effects of barometric pressure changes (the problems of the trapped gas expansion); (2) familiarize with the personal hypoxia symptoms and correct the hypoxia under the unpressurized environment; (3) learn the techniques to use the emergency oxygen system and portable oxygen equipment; (4) understand the degradation of night vision acuity affected by decreased oxygen. In this study, we used the self-reported questionnaire to obtain the data of hypoxia symptoms between the last and current altitude hypoxia trainings.

A structured questionnaire was developed to collect the information about the military helicopter aircrews’ hypoxia symptoms of the last and current altitude hypoxia trainings and the experience of in-flight hypoxia events. There were four sections of this questionnaire including (1) demographic information: age (<30, 30–39, ≥40 years), gender (male and female), role (pilot and non-pilot), flight years (<5, 5–9, 10–14, 15–19, ≥20 years), flight hours (<500, 500–999, 1000–1999, ≥2000 h), (2) self-reported hypoxia symptoms of the last and current altitude hypoxia trainings such as difficulty concentrating, cognitive impairment, visual impairment, tingling in extremities, paresthesia, hot flushes, numbness, air hunger, tiredness, dizziness/lightheadedness, anxiety, and nausea [12,13,14,15], (3) characteristics of in-flight hypoxia events: the experience of in-flight hypoxia during the flight career (no, yes), flight altitude [<4267 m (14,000 ft), ≧4267 m (14,000 ft)], how to detect the ongoing hypoxia event (personal hypoxia symptoms, abnormal sensation), flight performance impacted by hypoxia (none, mild degradation, significant degradation), and (4) the items of in-flight hypoxia symptoms similar to those in altitude hypoxia training.

### 2.3. Protocol

Military helicopter aircrews registered and attended a 5486-m (18,000-ft) refresher altitude hypoxia training at the APRL site in 2019. During the registration, aviation physiologists checked their certificates of annual medical examination and medical clearance and approved them fit for this training. We encouraged and recruited eligible subjects volunteered to participate this noninvasive survey. Eligible military helicopter aircrews that completed the paper-informed consent and agreed to participate into this study.

During the pre-training briefing, the APRL instructor introduced the purposes and procedures of the altitude chamber training for military helicopter aircrews. After that, the APRL instructor distributed the questionnaires to obtain the information of the experience with the “last” altitude hypoxia training (four years ago) and in-flight hypoxia events during the flight career in their recall memory.

**Figure 1:**
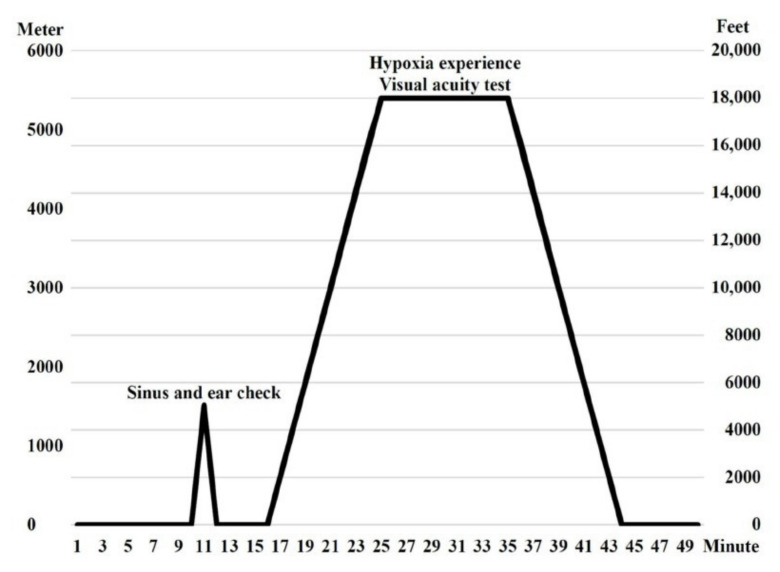
Profile of altitude hypoxia training.

Before the chamber ascent, mask fitting supplied 100% oxygen and communication check was tested by the APRL instructor and inside observers. At the beginning of the altitude hypoxia training, an ear and sinus check was conducted by ascending to 1524 m (5000 ft) and descending to ground level at a rate of 1524 m (5000 ft) per minute. Afterwards, the target altitude elevated to 5486 m (18,000 ft) at a rate of 610 m (2000 ft) per minute. At 5486 m (18,000 ft), trainees removed their oxygen mask and were exposed to mild hypoxia and the dim-light environment for 10 min. Trainees understood the hypoxia effect on the night visual acuity by using the visual test card and familiarized their personal hypoxia symptoms. Then, the 100% oxygen supply was resumed and returned to ground level at a rate of 610 m (2000 ft) per minute (Figure 1).

After the completion of the “current” altitude hypoxia training, trainees immediately reported their personal symptoms on the questionnaires again. Meanwhile, the instructor debriefed the whole chamber flight and responded to their questions.

### 2.4. Statistical Analyses

Demographic data of the study subjects, distribution of hypoxia symptoms and characteristics of the in-flight hypoxia events were descriptively displayed by the number and percentage for the subgroup of each variable. In the analytic test, the *McNemar* test was used to examine the consistency association of the hypoxia symptoms between the last and current altitude hypoxia trainings as well as between the last, and current altitude hypoxia trainings and in-flight hypoxia events. SPSS Statistic 24 software (IBM, Armonk, NY, USA) was applied to analyze the data collected from the eligible subjects. The level of statistical significance was accepted at the two-tailed 0.05 alpha level.

## 3. Results

For the demographic data shown in Table 1, there were 213 military helicopter aircrews completed the questionnaires in the study period. The proportion of age subgroups with less than 30, ranged from 30 to 39, and with more than 40 years were 10.8%, 52.6%, and 36.6%, respectively. There were only seven participants (3.3%) that involved female aircrews and 76.1% of them were pilots. About 40% of them had less than 10 flight years; 17.4%, 21.2%, and 18.3% of study subjects had 10–14, 15–19, and ≥20 years of flight experience. Almost two-thirds of those study subjects accumulated more than 1000 flight hours.

The five most commonly hypoxia symptoms of the last altitude hypoxia training were visual impairment (32.9%), difficulty concentrating (23.5%), cognitive impairment (19.7%), tiredness (16.9%), and air hunger (11.7%). Corresponding to these symptoms of the current altitude hypoxia training, the top five symptoms by ranking order were visual impairment (31.5%), tiredness (27.2%), difficulty concentrating (23.0%), cognitive impairment (14.1%), and air hunger (13.1%). The whole list of the 12 hypoxia symptoms mentioned can be seen in Table 2. Visual impairment (20.7%), difficulty concentrating (12.7%), tiredness (12.2%), cognitive impairment (8.0%), and air hunger (5.2%) were the five dominant symptoms that the subjects experienced both in the last and current altitude hypoxia trainings. Except for tiredness, the number of other symptoms reported were not significantly different between the last and current altitude hypoxia trainings.

Table 3 demonstrates the subgroup distribution of study subjects stratified by flight hours reported their symptoms of the last and current altitude hypoxia trainings. Among the young aircrews (<1000 flight hours), the five frequent symptoms of the last altitude hypoxia training were the same as those of the current altitude hypoxia training, and the listed hypoxia symptoms similar to the senior aircrews (≥1000 flight hours) were visual impairment, tiredness, difficulty concentrating, cognitive impairment, and air hunger by order. The percentage of the main symptoms occurred between those two groups was almost not significantly different between the last and current altitude hypoxia trainings.

As detailed in Table 4, eight aircrews (3.8%) claimed the experience of in-flight hypoxia. The flight altitude was lower than 4267 m (14,000 ft) during the onset of hypoxia event. Nearly two-thirds of the respondents detected the hypoxia symptoms similar to those they experienced during the previous altitude hypoxia training. Some cases unveiled that their flight performance was mildly (50.0%) or significantly (25.0%) degraded by hypoxia attack.

As shown in Table 5, we compared the frequency of the common hypoxia symptoms between the last, current altitude hypoxia trainings, and the in-flight hypoxia events. Of the eight aircrews, these main symptoms including difficulty concentrating, tiredness, visual disturbance, cognitive impairment, and air hunger, commonly occurred in the last, current altitude hypoxia training and during the flight. The occurrence rate of the common hypoxia symptoms of the last or current altitude hypoxia training were the same as those in-flight hypoxia events (all *p* values > 0.05).

## 4. Discussion

As summarized in this survey, there were eight in-flight hypoxia events (3.8%) sampling from the military helicopter aircrews who regularly accepted the altitude hypoxia training. Subjects were aware of ongoing hypoxia based on the former experience of altitude chamber flight. The advantage of this study was that we found the symptoms of the in-flight hypoxia events significantly consistent with those of the last and current altitude hypoxia trainings.

In Saudi Arabia, Smith (2007), identified that the main hypoxia related symptoms below 3048 m (10,000 ft) in a depressurized chamber were error prone, slowed responses, physical tiredness, difficulty thinking, and poor concentration [16]. In Canada, Bouak et al., unveiled that the physical hypoxic items of the military helicopter pilots were at a higher percentage at the resting status after the exposure of mild hypobaric hypoxic environment [17]. In the United States study, subjects indicated that the main low-grade hypoxic symptoms were sleepy and tired feeling, difficulty concentrating, light headed, eye irritation, and blurred vision at 3658 m (12,000 ft) [15]. Common hypoxia symptoms were impaired behavioral performance, cognitive function, visual interference, and unspecific sensation to pain noted at the altitude from 4572 m (15,000 ft) to 5486 m (18,000 ft) [18]. Among the military helicopter aircrews, our findings displayed the dominant hypoxia symptoms overlapped with those formerly reported were visual, cognitive impairment, difficulty concentrating, tiredness, and air hunger at this current exposure of 5486-m (18,000-ft) chamber flight. However, several researches identified that, the main hypoxia symptoms at the simulated altitude above 6096 m (20,000 ft) were mental impairment, coordination off, visual disturbance, lightheadedness/dizziness, and hot flushes [10,19]. There were two potential reasons to explain this slight disparity of dominant hypoxia symptoms between the different altitude exposures. First, stages of hypoxia based on the exposed altitude are classified into indifferent [<3048 m (<10,000 ft)], compensatory [3048–4572 m (10,000–15,000 ft)], disturbance [4572–6096 m (15,000–20,000 ft)], and critical stages [6096 m (>20,000 ft)] extracted from the US Army Aeromedical Training for Flight Personnel [18]. The altitude was 5486 m (18,000 ft) grouped into the critical stage in this work, being different from more than 6096 m (20,000 ft), belonged to the disturbance stage in other studies. Second, aircrews in the former studies learned that the hypoxia symptoms in the simulated altitude above 6096 m (20,000 ft), they were immediately trained to take the corrective procedure as soon as possible. However, the principle purpose of mild or moderate hypoxia training [below 5486 m (18,000 ft)] in this current study is to experience the diminished night vision and recognize the hypoxia symptoms lasting for 10 min. Therefore, trainees might detect different dominant symptoms depending on the training scenarios.

Researches summarized that the features of personal hypoxic symptoms gradually diminished with the time passed at the altitudes of the critical stages between the hypoxia awareness training sessions [3,8,11,20]. Repeated hypoxia training is mandatory to strengthen and deepen the memory of unique individual hypoxia signature [21]. A recent work also illustrated that the occurrence rate of hypoxia symptoms between the two chamber flights were not only different at the senior aircrew population who attended more times at the 7620-m (25,000-ft) chamber training [19]. In contrast to the former findings, this study data clarified that dominant hypoxia symptoms between the last and current altitude hypoxia trainings were almost non-different at the mild hypoxic altitude, even stratified by flight experience. The possible explanation might also come from the different training altitudes, exposure duration, or corrective steps inside the hypobaric chamber.

Characteristics of the helicopter or rotary airframes are unpressurized cabins and without oxygen supplement system. Aircrew members are actually exposed to the low oxygen concentration environment corresponding to the real flight altitude. Therefore, there are several restrictions of flight altitude and time to prevent the hypoxia hazard. In Taiwan, altitude of rotary aircrafts must be below 4267 m (14,000 ft) during flight based on the guidance of the Military Aviation Medicine Manual. Total flight time over 3048 m (10,000 ft) per sortie must be controlled to less than one hour [22]. However, Patrao et al., (2013) noted that there were approximately 40% of pilots reported the appearance of the symptoms even if the majority of time they flew below 3048 m (10,000 ft) [23]. Our results also noted that eight in-flight hypoxia events all happened at the heights below 14,000 feet. Those aircrews could not remember the exact flight time above 3048 m (10,000 ft). However, they ensured that the flight time were not over one hour because of the task features in Taiwan. In addition, Nishi et al., (2011) classified in-flight hypoxia cases into three groups [<1524 m (5000 ft), 1524–2438 m (5000–8000 ft), and >2438 m (8000 ft)]. The incidence rate of symptoms suggesting hypoxemia was 0.9%, 3.3%, and 25.4%, respectively; and increased by the altitude [13]. Due to the weakness of recalled memory in this study, we could not further calculate the frequency times of the in-flight hypoxia events grouped by the different altitudes. In line with the findings of the former few studies [15,16,17], this current study emphasized that the helicopter aircrews were still influenced and degraded their performance by hypoxia, even if the altitude didn’t surpass the physiological altitude during flight.

Previous researches have demonstrated that aircrews of fixed-wing airframes detected ongoing in-flight hypoxia incidents based on the memory of personal hypoxia symptoms and sensations inside the chamber flights [3,4,15,24,25]. Nevertheless, to the best of our knowledge, there are still no studies to investigate the consistent relationship between the altitude hypoxia training and the in-flight hypoxia events for helicopter aircrews. Our team extended the content to illustrate the tight associations between the last, current altitude hypoxia trainings and the in-flight hypoxia events. In other words, the helicopter aircrews also detected the ongoing in-flight hypoxia by using personal experience from the routine altitude hypoxia training. Although there were only a small number of in-flight hypoxia cases, the main contribution of this study is to highlight that the hypoxia threat during the flight should be more concerned among the helicopter aircrew population.

Some limitations were noted in this survey which collected the data by questionnaires. First, recall errors and reporting variation should exist in the present study. Second, a portion of the military helicopter aircrews were sampled to join this survey; thus, the generalization of these findings is limited. Third, we could not monitor the cardiovascular parameters (e.g., heart rate, oxygen saturation, etc.), and test the working function so as to better understand how the subjects’ performances were affected by the 10 min mild hypoxia exposure. Next, the effects of self-imposed factors and fatigue scales which increase the physiological altitude could not be traced back before the flight among those in-flight hypoxia events. Finally, the flight operation should be one type of physical exertion which consumes more oxygen and increases the severity of the hypoxia condition. However, the level of physical activity had not be able to be quantified for those events in this study. In the next work, a larger amount of study subjects will be recruited to increase the evidence power. Potential hypoxia-related factors and physiological parameters monitoring would be involved to provide more objective information.

## 5. Conclusions

In conclusion, the present study discovered that the dominant symptoms included visual impairment, difficulty concentrating, cognitive impairment, tiredness, and air hunger during the exposure of mild hypoxia conditions. We consequently determined a series of the consistency of hypoxia symptoms between the last, current altitude hypoxia training, and the in-flight hypoxia events among the helicopter aircrews. Thus, a refresher course of the hypoxia training is also important for the helicopter aircrews that usually fly below these physiological altitudes.

## Figures and Tables

**Table 1:** Demographic data of study subjects.

Variables	*n* (%)
Age (years)	
<30	23 (10.8%)
30–39	112 (52.6%)
≧40	78 (36.6%)
Gender	
Male	206 (96.7%)
Female	7 (3.3%)
Role	
Pilot	162 (76.1%)
Non-pilot	51 (23.9%)
Flight years	
<5	41 (19.2%)
5–9	51 (23.9%)
10–14	37 (17.4%)
15–19	45 (21.2%)
≧20	39 (18.3%)
Flight hours	
<500	45 (21.1%)
500–999	48 (22.5%)
1000–1999	73 (34.3%)
≧2000	47 (22.1%)

**Table 2:** Comparison of hypoxia symptoms between the last and current altitude hypoxia trainings.

Symptoms	Last *n* (%)	Current *n* (%)	Both * n (%)	*p* Value
Visual impairment	70 (32.9%)	67 (31.5%)	44 (20.7%)	0.775
Tiredness	36 (16.9%)	58 (27.2%)	26 (12.2%)	0.001
Difficulty concentrating	50 (23.5%)	49 (23.0%)	27 (12.7%)	1.000
Cognitive impairment	42 (19.7%)	30 (14.1%)	17 (8.0%)	0.073
Air hunger	25 (11.7%)	28 (13.1%)	11 (5.2%)	0.720
Hot flushes	12 (5.6%)	26 (12.2%)	9 (4.2%)	0.003
Dizziness/lightheadedness	15 (7.0%)	20 (9.4%)	8 (3.8%)	0.359
Anxiety	8 (3.8%)	14 (6.6%)	4 (1.9%)	0.180
Paresthesia	3 (1.4%)	9 (4.2%)	3 (1.4%)	0.031
Numbness	7 (3.3%)	6 (2.8%)	3 (1.4%)	1.000
Tingling in extremities	1 (0.5%)	4 (1.9%)	1 (0.5%)	0.250
Nausea	1 (0.5%)	3 (1.4%)	0 (0.0%)	-

*: Symptoms appeared both in the last and current altitude hypoxia trainings.

**Table 3:** Comparison of hypoxia symptoms between the last and current the altitude hypoxia trainings stratified by flight hours.

Symptoms	Last *n* (%)	Current *n* (%)	Both * n (%)	*p* Value
<1000 h (*n* = 93)				
Visual impairment	28 (30.1%)	25 (26.9%)	15 (16.1%)	0.678
Difficulty concentrating	22 (23.7%)	19 (20.4%)	8 (8.6%)	0.690
Cognitive impairment	20 (21.5%)	11 (11.8%)	7 (7.5%)	0.049
Tiredness	20 (21.5%)	30 (32.3%)	13 (14.0%)	0.064
Air hunger	12 (12.9%)	13 (14.0%)	5 (5.4%)	1.000
≥1000 h (*n* = 120)				
Visual impairment	42 (35.0%)	42 (35.0%)	29 (24.2%)	1.000
Difficulty concentrating	28 (23.3%)	30 (25.0%)	19 (15.8%)	0.824
Cognitive impairment	22 (18.3%)	19 (15.8%)	10 (8.3%)	0.664
Tiredness	16 (13.3%)	28 (23.3%)	13 (10.8%)	0.008
Air hunger	13 (10.8%)	15 (12.5%)	6 (5.0%)	0.804

*: Symptoms appeared both in the last and current altitude hypoxia trainings.

**Table 4:** Description of in-flight hypoxia events.

Variables	*n* (%)
In-flight hypoxia	
No	205 (96.2%)
Yes	8 (3.8%)
Altitude	
<4267 m (14,000 ft)	8 (100.0%)
≥4267 m (14,000 ft)	0 (0.0%)
Detection	
Personal hypoxia symptoms	5 (62.5%)
Abnormal sensation	3 (37.5%)
Impact of flight performance	
None	2 (25.0%)
Mild degradation	4 (50.0%)
Significant degradation	2 (25.0%)

**Table 5:** Consistency relations of hypoxia symptoms between the last, current altitude hypoxia trainings and in-flight hypoxia events.

Symptoms	Both of Last and In-Flight * n (%)	*p* Value	Both of Current and In-Flight ^§^ *n* (%)	*p* Value
Visual impairment	2 (25.0%)	1.000	2 (25.0%)	0.500
Cognitive impairment	2 (25.0%)	1.000	3 (37.5%)	1.000
Difficulty concentrating	2 (25.0%)	1.000	3 (37.5%)	0.250
Tiredness	3 (37.5%)	1.000	3 (37.5%)	0.375
Air hunger	1 (12.5%)	1.000	1 (12.5%)	1.000

*: Symptoms appeared both in the last altitude hypoxia training and the in-flight hypoxia events. **^§^** : Symptoms appeared both in the current altitude hypoxia training and the in-flight hypoxia events.

## Data Availability

The data in this study are collected and owned by Aviation Physiology Research Laboratory, Kaohsiung Armed Forces General Hospital, Gangshan Branch, Taiwan. Due to the regulations, the data cannot be shared publicly.
